# P-353. *Aspergillus* Burdens in Outdoor and Indoor Air at Hospitals Housing Patients at High Risk for Invasive Fungal Infections

**DOI:** 10.1093/ofid/ofae631.554

**Published:** 2025-01-29

**Authors:** Zachary Wilkins, Seong-Yoon Cho, Binghua Hao, Eileen Driscoll, Shaoji Cheng, Giuseppe Fleres, Alexander Sundermann, Ashley Ayres, Graham M Snyder, Minh-Hong Nguyen, Cornelius J Clancy

**Affiliations:** University of Pittsburgh, Pittsburgh, Pennsylvania; University of Pittsburgh, Pittsburgh, Pennsylvania; University of Pittsburgh Medical Center, Pittsburgh, Pennsylvania; University of Pittsburgh, Pittsburgh, Pennsylvania; University of Pittsburgh, Pittsburgh, Pennsylvania; University of Pittsburgh, Pittsburgh, Pennsylvania; University of Pittsburgh, Pittsburgh, Pennsylvania; UPMC, Pittsburgh, Pennsylvania; University of Pittsburgh, Pittsburgh, Pennsylvania; University of Pittsburgh, Pittsburgh, Pennsylvania; University of Pittsburgh, Pittsburgh, Pennsylvania

## Abstract

**Background:**

Airborne dissemination of conidia is implicated in transmission of pathogenic fungi. There is lack of knowledge on airborne fungal burdens in hospital environments.

**Figure:**
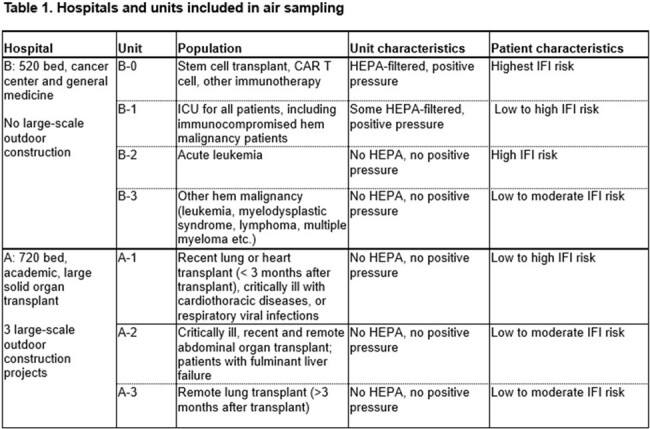

**Methods:**

Air at hospitals A and B (1.5 miles apart) was sampled monthly (SASS 3100; 36000 L/2 hrs) from May ‘23-April ‘24 including outdoor, lobby, nurse stations (NS) and patient (pt) rooms on units caring for patients with immunocompromising conditions [Table 1]. New building construction was ongoing next to hospital A. Filter extracted DNA genome equivalent (GE) was quantitated by pan-*Aspergillus* (ASP) qPCR and standard curve; sonicated and non-sonicated filters were cultured. Descriptive statistics, Mann-Whitney U and Wilcoxon tests were used.

**Figure 1.**
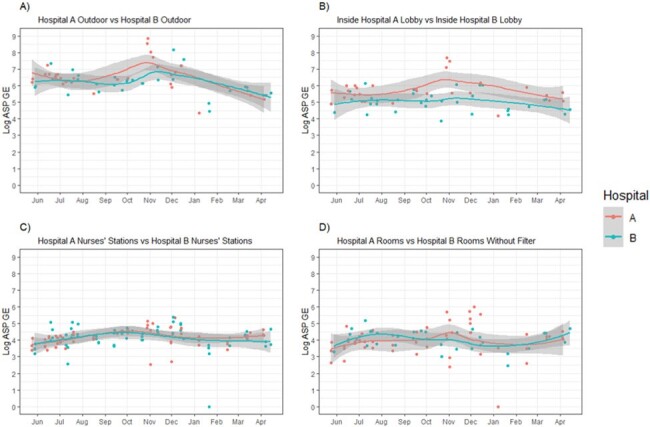
Comparisons of Aspergillus burdens at 2 hospitals 1A. Comparison of log ASP GE of outdoor air (outside of the entrance to the hospital lobby) over time at hospitals A and B (standard error shown in grey shade). 1B. Log ASP GE of air in the lobby of hospitals A and B. Note that ASP burden was higher in the lobby of hospital A, which is much more heavily trafficked than the lobby of hospital B. 1C. Log ASP GE of air at the nurses’ stations of 7 units in hospitals A and B. There is no significant difference between burdens at the 2 hospitals (p=0.74). 1D. Log ASP GE for rooms at hospitals A and B without HEPA filtration and positive pressure. Curves were plotted via locally estimated scatterplot smoothing (LOESS) with standard error shown in grey.

**Results:**

Mean log-GEs were greatest for outdoor samples, followed by lobby, NS and pt rooms. Protected (HEPA-filtered and positive pressure) pt room GEs were significantly lower than unprotected rooms. There were no significant location-specific differences between hospitals A and B, except the lobbies [Fig 1]. GE from HEPA filtered rooms in hospital B unit 0 was taken as a comparative “safety threshold”, since filtering protects against infections. Extra GE burden in unprotected rooms was ∼0.5-1.2 log GE. A construction accident that exposed a unit in hospital A directly to outdoor air caused a ≥ 1-log GE spike that persisted from 2-4 weeks [Fig 2]. Seasonality data indicate outdoor peak and trough GEs in fall and spring, respectively. GEs were validated by + ASP and *A. fumigatus* air cultures [Fig 3].

**Figure 2.**
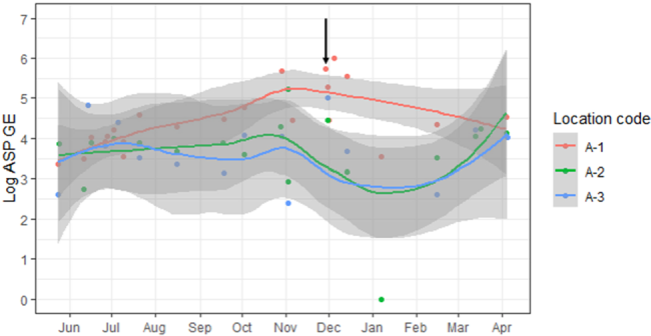
Trends of Aspergillus burden in units at hospital A

Trends of log ASP GE over time for the patients’ rooms at hospital A ( A-1, A-2, A-3 units). Three new buildings were under construction within a block of hospital A. A-1 is the unit closest to the construction. Note the higher ASP burden in A-1 rooms compared with other units. Breach of the wall separating the outside environment and the patients’ rooms in A-1 occurred in late Nov (arrow). Note 1) the higher ASP GE in unit A-1 than other units even before the accident, and 2) the further increase in burden around the accident, which gradually came down to baseline after 2-3 weeks. Curves were plotted via LOESS with standard error shown in grey.

**Conclusion:**

Airborne fungal exposure within hospitals is significantly lower than immediately outside the hospital. Fungal burdens within protected pt rooms can be used to estimate extra burdens encountered by immunosuppressed pts in unprotected rooms. Elevated fungal burdens stemming from contamination of a unit can persist for weeks following remediation. We are continuing studies of seasonality and correlations between airborne burdens, environmental and clinical fungal cultures, and fungal infections.

**Figure 3.**
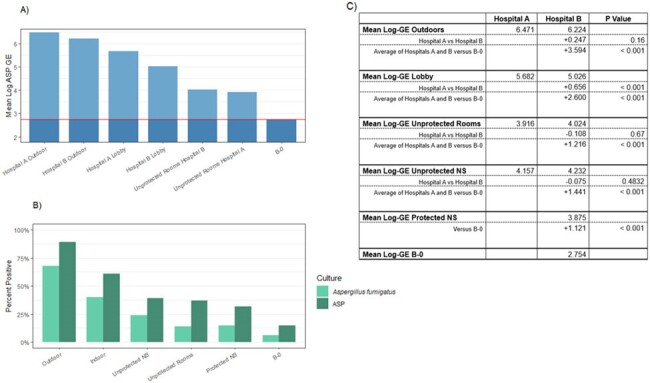
Comparison of Aspergillus burden measured by qPCR and culture, stratified by locations 3A. Mean log ASP GE for each location sampled. The reference line is the difference in mean log ASP GE when comparing that location to the B-0 rooms (HEPA-filtered, positive pressure). Note that the differences in log ASP GE between B-0 and other unfiltered units range from 0.52 to 1.2-log ASP GE. Upon entering the lobby, the log ASP GE was decreased by a median of ∼1 log, which was further decreased in patients’ rooms (3.16 to 4.42). 3B. Percent of positive cultures from each sampled location. AF designates cultures positive for Aspergillus fumigatus while ASP designates pan-Aspergillus¬ cultures. There was ∼30% decrease in positive cultures from outdoors to indoors, and an additional >10% decrease when moving to units. Culture positivity in unprotected NS and rooms was higher than protected NS and rooms by ∼10%. 3C. There is no significant difference between the means of GEs between the outdoors of the 2 hospitals (p=0.16). ASP burden was higher in the lobby of hospital A than B (p<0.001). There was no significant difference between means of the log ASP GE between the unfiltered rooms at hospital A and the unfiltered rooms at hospital B (p=0.67).

**Disclosures:**

**Alexander Sundermann, DrPH, CIC, FAPIC**, OpGen: Honoraria **Graham M. Snyder, MD, SM**, Infectious Diseases Connect: Advisor/Consultant **Cornelius J. Clancy, MD**, Cidara: Grant/Research Support|Gilead: Honoraria|Merck: Grant/Research Support|Scynexis: Advisor/Consultant|Shionogi: Advisor/Consultant|Venatorx: Advisor/Consultant

